# Commentary: The Role of the Anion in Salt (NaCl) Detection by Mouse Taste Buds

**DOI:** 10.3389/fncel.2019.00502

**Published:** 2019-11-08

**Authors:** Vaibhav Menon, Yu-Chieh David Chen

**Affiliations:** Interdepartmental Neuroscience Program, University of California, Riverside, Riverside, CA, United States

**Keywords:** salt, taste transduction, anion, taste cells, fungiform, amiloride-insensitive

Among the five basic taste qualities, much is known about the molecular receptors and corresponding taste receptor cell types in rodent taste buds for sweet, bitter, umami, sour, and low salt taste (Chandrashekar et al., 2010; Liman et al., 2014; Roper and Chaudhari, 2017; Tu et al., 2018). In contrast, the molecular and cellular basis of high salt taste is incompletely understood, despite the known physiological importance of salt in regulating water homeostasis, ion concentration gradients, and sodium appetite (Schulkin, 1991). In addition, excessive sodium consumption in dietary food intake has been linked with hypertension and cardiovascular diseases (Aaron and Sanders, 2013), underscoring the importance of elucidating the high salt transduction pathway and its major molecular components.

Taste buds, the functional units of taste transduction, are located in three morphologically distinct gustatory papillae (circumvallate, foliate, and fungiform) in different parts of the rodent tongue and the palate, which are innervated by different peripheral nerves. There are three morphological types of taste receptor cells (TRCs) in taste buds (Liman et al., 2014; Roper and Chaudhari, 2017): Type 1 TRCs are glial-like cells; Type 2 TRCs express T1R or T2R receptors for detection of sweet, bitter, and umami substances; and Type 3 TRCs express the Otopetrin, a candidate proton channel mediating sour taste. Previous studies have recorded salt responses in the chorda tympani nerve (Hill et al., 1982), which conveys input from the fungiform papilla in the anterior part of the tongue. Depending on its concentration, salt (NaCl) elicits two distinct behavioral responses: appetitive low concentration (<100 mM) and aversive high concentration (>300 mM) responses. Two distinct salt transduction mechanisms have been reported, separable by sensitivity to the diuretic amiloride. The first, an amiloride-sensitive pathway, is Na^+^-selective for low salt detection, with epithelial sodium channel ENaC identified as the underlying molecular receptor for mediating low-salt attraction (Vandenbeuch et al., 2008; Yoshida et al., 2009; Chandrashekar et al., 2010). The second, an amiloride-insensitive pathway, is cation non-selective for high salt detection. Notably, the amiloride-insensitive response component is the major contributor (~80%) of the overall high salt responses recorded in the chorda tympani nerve in mice (Lu et al., 2016). However, the underlying transduction mechanism and the identity of taste cell types involved in both pathways remain elusive. A recent article in the Journal of Neuroscience by Roebber et al. (2019) reports that amiloride-insensitive high salt transduction occurs in Type 2 TRCs and is largely dependent on the presence of the Cl^−^ anion.

Physiological measurement of salt responses is complex and challenging given that both the cation (Na^+^) and anion (Cl^−^) are present in extracellular fluids, and the application of hypertonic/hypotonic stimuli to the entire extracellular space may induce non-physiological cellular changes. To circumvent these problems, Roebber et al. (2019) performed *ex vivo* Ca^2+^ imaging and recorded cellular responses of different taste cell types in a semi-intact preparation of mouse taste buds. This method allows precise spatiotemporal control of tastant delivery to the apical tips of the taste bud cells while maintaining the same ionic concentration in the rest of the cell bodies. By mimicking the *in vivo* stimulation with salt, the authors reported robust concentration-dependent cellular responses to NaCl that were amiloride-insensitive. Prolonged stimulation with NaCl resulted in rapid adaptation of Ca^2+^ responses (Roebber et al., 2019), consistent with previously described electrophysiological recordings of chorda tympani nerve responses to NaCl (Beidler, 1953).

Importantly, Roebber et al. (2019) provided several lines of evidence that it is the Type 2 but not Type 3 TRCs that are involved in the amiloride-insensitive responses to high salt. First, *post-hoc* immunohistochemistry staining showed that TRCs with amiloride-insensitive high salt responses were co-labeled with antibodies against a Type 2 TRC marker (anti-PLCß2) but not a Type 3 TRC marker (anti-Car4). Second, TRCs with amiloride-insensitive high salt responses did not require external Ca^2+^, characteristic of Type 2 TRCs that utilize internal Ca^2+^ stores as opposed to Type 3 TRCs that express voltage-gated Ca^2+^ channels to allow entry of external Ca^2+^. Finally, sequential application of NaCl and other diagnostic tastants to the same taste bud showed a broad overlap in populations of cells that responded to both NaCl and to saccharin or a bitter mixture of cycloheximide and denatonium, which are known to activate Type 2 TRCs. This result is consistent with a previous study showing that bitter-sensing Type 2 TRCs also exhibit amiloride-insensitive high salt responses (Oka et al., 2013). Since saccharin activates both sweet and bitter taste receptors (Kuhn et al., 2004), it is unclear whether the Type 2 TRCs that respond to both NaCl and saccharin represent bitter TRCs or whether they comprise both sweet and bitter TRCs. It has been proposed that high salt co-opts the bitter transduction pathway to ensure robust behavioral aversion (Oka et al., 2013); however, a recent study in *Drosophila* reported that peripheral gustatory sweet-sensing neurons are also activated by high concentrations of NaCl (Jaeger et al., 2018). An intriguing parallel is that mammalian TRCs for appetitive tastes, such as sweet or umami, might also respond to high salt. Consistent with this possibility, Roebber et al. (2019) found that 9 out of 35 imaged TRCs responded only to high salt but not to saccharin or the bitter mixture, suggesting that they may be Type 2 TRCs for umami taste. Future experiments with imaging the same taste bud with sequential application of NaCl and either sweet or umami substances would clarify the involvement of each subset of Type 2 TRCs in high salt responses.

In contrast, the authors showed that citric acid, a tastant known to activate Type 3 TRCs, did not activate any cells that responded to NaCl, consistent with previous Ca^2+^ imaging results that found distinct populations of TRCs responding to high salt and sour stimuli (Chandrashekar et al., 2010). Intriguingly, another study reported that blocking synaptic transmission of Type 3 sour cells (PKD2L1-expressing cells) by expression of the tetanus toxin light chain reduced amiloride-insensitive high salt responses in the chorda tympani nerve, indicating the role of Type 3 sour cells in high salt detection (Oka et al., 2013). Given the lack of functional imaging evidence for overlap of high salt and sour receptivity in the taste buds, one possibility is that rather than acting as direct high salt sensors, Type 3 sour cells modulate high salt responses through interaction with other types of TRCs via gap junctions or paracrine signaling (Roper and Chaudhari, 2017). Taken together, the results provided by Roebber et al. (2019) strengthen the idea that Type 2 TRCs are the cellular substrates for amiloride-insensitive high salt responses in fungiform papilla.

To explore the molecular mechanism underlying the amiloride-insensitive high salt responses in fungiform papilla, the authors substituted choline chloride for NaCl and found that choline chloride yielded Ca^2+^ responses comparable to those of NaCl in both the presence and absence of extracellular Na^+^. In addition, when Na-gluconate was substituted for NaCl, Ca^2+^ responses were totally abolished, suggesting Cl^−^ but not Na^+^ plays a major role in amiloride-insensitive high salt responses (Roebber et al., 2019). Although Cl^−^ is essential for high salt responses, a broader role of the anion in high salt responses cannot be ruled out since NaBr elicited a partial response as compared to NaCl, consistent with an ability of other anions to elicit high salt responses. This may explain why the authors could not identify the molecular machinery underlying high salt transduction using a candidate gene approach targeting Cl^−^ channels or transporters with pharmacological inhibitors and ion substitution experiments (Roebber et al., 2019). In addition, the contribution of the cation remains unclear since substituting KCl for NaCl reduced high salt responses by 50% (Roebber et al., 2019). It is possible that K^+^, but not Na^+^, interferes with unidentified anion-mediated salt transduction machinery, conceivably anion-selective channels such as Pannexin 1 or Cystic fibrosis transmembrane conductance regulator (CFTR), which are known to be expressed in rodent taste buds (Huang et al., 2007; Merigo et al., 2008; Ma et al., 2012; Hwang and Kirk, 2013). Identifying the molecular underpinnings of anion-mediated salt transduction would provide further insight into how K^+^ interacts with this pathway.

In conclusion, Roebber et al. (2019) demonstrated that Type 2 TRCs in fungiform papillae mediate amiloride-insensitive high salt responses that are predominantly driven by the Cl^−^ anion. This work provides an important step toward the full picture of how different concentrations of salts are differentially encoded in the gustatory system. Together with prior findings that amiloride-insensitive high salt responses were observed in Type 3 TRCs of the circumvallate papillae (Ninomiya, 1998; Lewandowski et al., 2016), it is intriguing that salt taste coding varies across different types of TRCs in anatomically and functionally distinct gustatory papillae (Figure 1). Future studies to investigate molecular machinery underlying individual salt transduction pathways, and mechanisms by which animals integrate salt-sensing inputs to generate opposite behavioral responses to low salt and high salt would inform strategies to reduce overconsumption of dietary salt.

**Figure 1:**
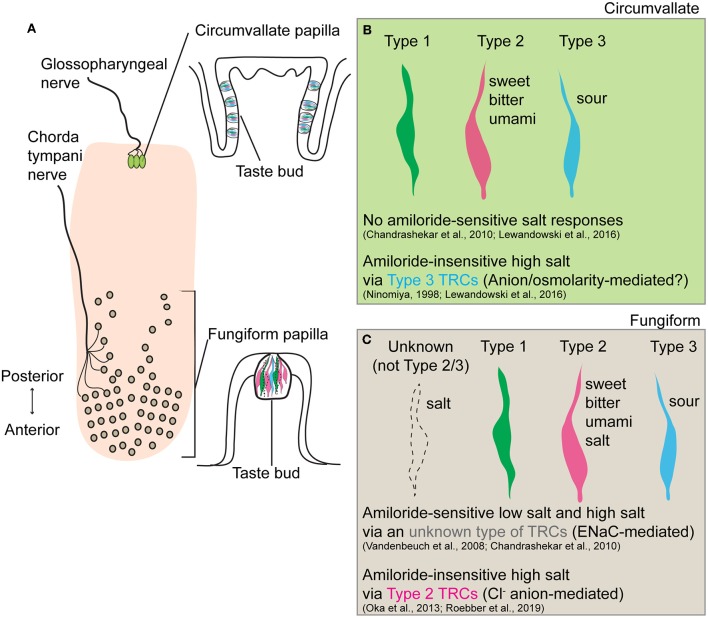
Schematic summary of salt taste transduction in circumvallate and fungiform papilla. **(A)** Anatomical location and morphology of two gustatory papillae (circumvallate and fungiform) in the rodent tongue. Taste inputs from circumvallate and fungiform papilla are transmitted through the glossopharyngeal and chorda tympani nerves, respectively. **(B)** In circumvallate papilla, the amiloride-insensitive high salt taste transduction is mediated in Type 3 TRCs, known sour taste transducers, through either an anion-selective or osmolality-sensitive channel. No amiloride-sensitive salt responses have been observed. **(C)** In fungiform papilla, the amiloride-sensitive low salt and high salt response is mediated through epithelial sodium channel ENaC in an unknown type of TRCs (neither Type 2 nor Type 3). Since Type 1 TRCs are the only cells sensitive to amiloride, these cells should be the cellular substrates of the ENaC-mediated salt taste. Also, Roebber et al. (2019) demonstrated that amiloride-insensitive high salt response is mediated by Type 2 TRCs, known sweet, bitter, and umami taste transducers, in a largely anion (Cl^−^) dependent manner. The specific receptor or transporter involved has yet to be identified.

## Author Contributions

VM and Y-CC contributed ideas and wrote the final manuscript.

### Conflict of Interest

The authors declare that the research was conducted in the absence of any commercial or financial relationships that could be construed as a potential conflict of interest.
